# Application of a Sociotechnical Framework to Uncover Factors That Influence Effective User Engagement With Digital Mental Health Tools in Clinical Care Contexts: Scoping Review

**DOI:** 10.2196/67820

**Published:** 2025-04-28

**Authors:** Brian Lo, Keri Durocher, Rebecca Charow, Sarah Kimball, Quynh Pham, Sanjeev Sockalingam, David Wiljer, Gillian Strudwick

**Affiliations:** 1 Centre for Addiction and Mental Health Toronto, ON Canada; 2 Institute for Health Policy, Management and Evaluation University of Toronto Toronto, ON Canada; 3 Arthur Labatt Family School of Nursing Western University London, ON Canada; 4 University Health Network Toronto, ON Canada; 5 Temerty Faculty of Medicine University of Toronto Toronto, ON Canada

**Keywords:** user engagement, nursing informatics, clinical informatics, mental health, clinical care settings

## Abstract

**Background:**

Digital health tools such as mobile apps and patient portals continue to be embedded in clinical care pathways to enhance mental health care delivery and achieve the quintuple aim of improving patient experience, population health, care team well-being, health care costs, and equity. However, a key issue that has greatly hindered the value of these tools is the suboptimal user engagement by patients and families. With only a small fraction of users staying engaged over time, there is a great need to better understand the factors that influence user engagement with digital mental health tools in clinical care settings.

**Objective:**

This review aims to identify the factors relevant to user engagement with digital mental health tools in clinical care settings using a sociotechnical approach.

**Methods:**

A scoping review methodology was used to identify the relevant factors from the literature. Five academic databases (MEDLINE, Embase, CINAHL, Web of Science, and PsycINFO) were searched to identify pertinent articles using key terms related to user engagement, mental health, and digital health tools. The abstracts were screened independently by 2 reviewers, and data were extracted using a standardized data extraction form. Articles were included if the digital mental health tool had at least 1 patient-facing component and 1 clinician-facing component, and at least one of the objectives of the article was to examine user engagement with the tool. An established sociotechnical framework developed by Sittig and Singh was used to inform the mapping and analysis of the factors.

**Results:**

The database search identified 136 articles for inclusion in the analysis. Of these 136 articles, 84 (61.8%) were published in the last 5 years, 47 (34.6%) were from the United States, and 23 (16.9%) were from the United Kingdom. With regard to examining user engagement, the majority of the articles (95/136, 69.9%) used a qualitative approach to understand engagement. From these articles, 26 factors were identified across 7 categories of the established sociotechnical framework. These ranged from technology-focused factors (eg, the modality of the tool) and the clinical environment (eg, alignment with clinical workflows) to system-level issues (eg, reimbursement for physician use of the digital tool with patients).

**Conclusions:**

On the basis of the factors identified in this review, we have uncovered how the tool, individuals, the clinical environment, and the health system may influence user engagement with digital mental health tools for clinical care. Future work should focus on validating and identifying a core set of essential factors for user engagement with digital mental health tools in clinical care environments. Moreover, exploring strategies for improving user engagement through these factors would be useful for health care leaders and clinicians interested in using digital health tools in care.

## Introduction

### Background

Digital mental health tools continue to have a critical role in advancing the delivery of mental health care in clinical care settings [1-4]. In 2020, the World Health Organization released a global strategy on digital health innovation [5], which advocated for the opportunities and need to advance the implementation of digital health strategies and people-centric health systems fueled by digital health tools. In the United States, the American Medical Association [6] found that physician adoption of telehealth during the COVID-19 pandemic grew 5-fold, and the use of remote monitoring tools doubled due to improvements in patient outcomes and work efficiency.

As such, many organizations have advocated for the use of digital health tools in mental health care delivery [7-9]; for example, the American Psychiatric Association has released a set of tool kits designed to support telepsychiatry and clinician recommendation of digital tools in the clinical setting [10], while the Mental Health Commission of Canada has released a number of tool kits to support adoption within Canada, including one for implementing digital mental health innovations [4]. There are also some frameworks for characterizing various digital mental health tools and use cases where digital tools can be helpful to support care [4]. Finally, the National Health Service in England has released a mental health digital playbook that focuses on outlining clinical care pathways that embed the use of digital mental health tools, as well as the associated governance, policies and change management [11]. This playbook, among others, has led to the uptake of many digital mental health tools across health systems, including Big White Wall (now known as Togetherall) in Canada and the United Kingdom [12-14] and the Digital Opportunities for Outcomes in Recovery Services program in the United States [15-19].

However, there continues to be limited evidence on the outcomes and impact of digital mental health tools within clinical care settings in real-world environments [20]. A recent systematic review synthesized findings from 19 trials on digital mental health apps and determined that there is inconclusive evidence to suggest that these tools can be recommended as stand-alone interventions [21]. Many challenges have hindered the effective adoption and utility of the tools. While some are related to user-centered design and the adaptation of evidence-based principles and content within the platform, an emerging area of concern is suboptimal engagement with the tool by patients and families [22]. Several studies have found that continued use of the tool rapidly decreased after initial use [22-24]. The results of a systematic review that looked at user engagement for 7 apps for depression and anxiety showed that <42% of users stayed engaged and continued to use the tool beyond 4 weeks [25]. To ensure that the expected benefits are realized for the end user, it is essential that there is sufficient user engagement where appropriate and necessary.

### User Engagement

User engagement has been a growing area of interest over the last few years, given the proliferation of tools and the recognition of this gap in the 2010s [22]. As a result, there have been numerous conceptualizations and characterizations of user engagement; for example, in 2008, O’Brien and Toms [26] characterized user engagement as a process that involves engagement and disengagement, and Perski et al [27] developed an integrative definition: “(1) the extent (e.g. amount, frequency, duration, depth) of usage and (2) a subjective experience characterised by attention, interest and affect.” Some work has also been conducted to characterize user engagement and to help visualize user engagement through use log data, Pham et al [28] developed a framework of metrics, while Pham et al [29] built an analytics platform. Likewise, Yaeger et al [30] looked at factors related to user engagement with a trauma recovery eHealth intervention using the Health Action Process Approach, while MacPhail et al [31] used the Health Action Process Approach to explore health behavior engagement in individuals with type 2 diabetes mellitus. Several researchers have since attempted to uncover factors related to user engagement in the context of facilitators and barriers [32], as well as neuropsychological [33] and persuasive design [34] frameworks. This process has resulted in a myriad of factors, from gamification and technical issues to personalization [32-34], alongside studies evaluating the impact of these interventions on boosting engagement [35].

However, to date, there have been very limited discussions on the factors that influence user engagement with digital tools used specifically in clinical care models and settings. While the majority of tools being developed are focused on self-help in the community [21], there is growing demand and interest from the clinical community in implementing digital tools in clinical care; for example, the Stepped Care Model 2.0 offered by the Mental Health Commission of Canada highlights how tools can be used to augment care being delivered for individuals across various care levels [36]. Given that the integration of digital tools requires careful consideration of the environment and the broader health system, there is a timely need to look at the factors relevant to user engagement with digital mental health tools used specifically in clinical care pathways and delivery.

### Sociotechnical Frameworks

One approach to addressing this issue is the application of a sociotechnical framework, which allows for the characterization and examination of complex environments to support the adoption and use of digital tools in clinical care settings [37]. In particular, it encourages the researcher to look at the interactions across components at the microlevel (eg, individual), mesolevel (eg, organizational), and macrolevel (eg, health system). The majority of digital health research has focused on factors related to end users or the tool itself [24]; as a result, it has yielded limited value in terms of understanding user engagement within the complex clinical environment. Applying a sociotechnical framework can help highlight the processes and workflows of the clinical environment as well as the broader policies of the organization and health system [38,39].

Frameworks that have been used to look at innovations in health care include the nonadoption, abandonment, scale-up, spread, and sustainability framework [40] and the sociotechnical framework developed by Singh and Sittig [41]. Greenhalgh et al [40] developed the nonadoption, abandonment, scale-up, spread, and sustainability framework, which focuses on examining features across the condition, technology, adopters, and organization, among others [40]. Similarly, the sociotechnical framework developed by Singh and Sittig [41] outlines 8 components that are focused across the micro-, meso-, and macrolevel factors. These eight components include (1) hardware and software computing infrastructure; (2) clinical content; (3) human-computer interface; (4) people; (5) workflow and communication; (6) internal organizational policies, procedures, and culture; (7) external rules, regulations, and pressures; and (8) system measurement and monitoring. Both frameworks have been used to examine various clinical innovations such as virtual care [38] and issues related to ransomware attacks [42]. In particular, they have been used to look at factors influencing the adoption of artificial intelligence in Canadian health care [43] and clinical handoff tools [44].

In this regard, this study aims to develop a comprehensive understanding of the factors related to user engagement with digital mental health tools in clinical care settings through the use of the sociotechnical framework developed by Singh and Sittig [41]. Obtaining a snapshot of the current evidence can help identify the current gaps in literature and inform the development of a comprehensive framework for assessing user engagement with digital mental health tools in clinical care contexts.

## Methods

### Overview

To identify the factors that influence user engagement with digital mental health tools in clinical care settings, we used a scoping review approach [45]. Given the exploratory nature and understanding of the factors related to user engagement, this approach was considered appropriate to identify a preliminary set of factors for further exploration [42]. The approach outlined by Arksey and O’Malley [45] and later refined by Levac et al [46] and Peters et al [47] was used. One of the authors (SK), a patient partner, was engaged in the development, implementation, and analysis of this scoping review.

### Step 1: Identify the Research Questions and Objectives

The research questions (RQs) of the scoping review are as follows:

- RQ1: Of the digital mental health tools being used in clinical care settings, what are the types of technologies (eg, mobile app and wearable) and functionalities used to deliver digital mental health care?
- RQ2: What are the characteristics of the populations that are using digital mental health tools as part of clinical care?
- RQ3: What are the sociotechnical factors that influence user engagement with digital mental health tools in clinical care environments over time?
- RQ4: What are the characteristics of clinical programs that embed digital mental health tools as a component of care?

We used the sociotechnical framework developed by Sittig and Singh [41] to guide the synthesis of factors that influence user engagement with digital mental health tools in clinical care environments. This ensured a comprehensive overview of the factors across individual, organizational, and health system levels.

### Step 2: Search Strategy Creation

To identify articles on user engagement with digital mental health tools in clinical care contexts, a systematic search strategy was developed based on previous search strategies [32,48]. Relevant Medical Subject Headings (MeSH) terms and keywords related to *user engagement*, *mental health,* and *digital health tools* were applied across databases (Multimedia Appendix 1). The search was conducted on 5 databases—MEDLINE, Embase, PsycINFO, CINAHL, and Web of Science—in January 2022 without restrictions on date or study type. These databases are popular among health sciences researchers and were expected to index most of the literature published in this field. The search strategy was first developed in MEDLINE and was adapted to other databases. A research librarian was also consulted in the refinement of the search strategy. The search strategy was validated by confirming whether previously identified relevant articles (eg, the study by Hoffman et al [17]) were included in the search. The search was updated in October 2023 using the same strategy and approach.

### Step 3: Selection of Studies

The inclusion and exclusion criteria for the scoping review are outlined in Textbox 1. Eligible studies must examine a digital health tool (eg, mobile app, patient portal, or wearable) that primarily addresses a mental health or addiction issue. To ensure relevance to clinical care, the tool must include at least 1 patient-facing component (eg, app) and 1 clinician-facing component (eg, dashboard) as specified in the Mental Health Commission of Canada Toolkit for E-Mental Health Implementation [4]. Studies must explore the concept of user engagement as an objective, following the aforementioned definition by Perski et al [27]. Articles published in languages other than English were excluded for feasibility. Systematic and literature reviews were excluded, but their reference lists were examined. Non–peer-reviewed article types, such as theses and conference presentations, were also excluded.

**Inclusion and exclusion criteria for the scoping review.**

**Inclusion criteria**

- Digital health tool must primarily address a mental health or addiction issue
- Digital health tool must include at least 1 patient-facing component and 1 clinician-facing component [5]
- Study must examine user engagement as per the definition by Perski et al [27]

**Exclusion criteria**

- Digital health tool is used by the patients themselves (eg, a self-help tool) and does not contain a clinician-facing component
- Article is not in English
- Study does not examine user engagement as an objective

Study selection was conducted by the first author (BL) in duplicate with 2 doctoral students (KD and RC) in health informatics using Covidence (Veritas Health Innovation Ltd). Deduplication was performed by identifying records with identical titles and publication years, with verification by a member of the project team (BL). Screening was carried out in 2 stages: an initial title and abstract review, followed by full-text screening of studies meeting the inclusion criteria. To ensure consistency, a pilot screening (n=100) of titles and abstracts was conducted with each of the two doctoral students (KD and RC). Any discrepancies were discussed and resolved by the 3 reviewers, and Cohen κ was used to assess interrater reliability [49]. A Cohen κ value of >0.70 was achieved in the pilot, after which we proceeded with the screening process. A similar process was followed for full-text screening, in that small pilot rounds were conducted by 2 doctoral students, and any discrepancies were discussed and resolved by the 3 reviewers.

### Step 4: Extracting and Charting the Data

The elements extracted for each RQ are outlined in Textbox 2. The article type, year of publication, and study objective were collected to understand the characteristics of the included articles. For RQ1, relevant information about the digital mental health tool, such as technology type, objective, and main features, were extracted. For RQ2—identifying the characteristics of the population—we collected information about the demographic characteristics of the users and access requirements for the tool. For RQ3, factors relevant to user engagement with digital mental health tools in clinical care contexts were extracted. Finally, for RQ4, information about the objective and the digital health delivery model was collected. KD and RC conducted a pilot extraction of 5 to 10 articles, and the extracted data were compared. On the basis of the feedback, the extraction table was iteratively refined (eg, by adding relevant elements).

**Data elements extracted (including for each research question [RQ]).**

**Characteristics of included articles**

- Year of publication
- Article type
- Country of publication
- Type of user engagement examined (subjective vs objective)
- Main objective of paper
- Methodology (eg, study design and type of measurements and instruments used)

**RQ1: Type of digital mental health tool used**

- Name of digital health tool
- Technology used for digital mental health tool
- Main objective of digital health tool
- Patient- and clinician-facing functionalities of the tool as per the Mental Health Commission of Canada Toolkit for E-Mental Health Implementation [4]

**RQ2: Population using digital mental health tool**

- Participant population
- Demographics of the participants in the study (eg, mental health condition)
- Duration of participation

**RQ3: Factors that influence user engagement with digital mental health tools**

- Factors relevant to user engagement with digital mental health tools in clinical care contexts

**RQ4: Clinical digital health delivery model**

- Objective of the treatment provided as part of the clinical care model
- Delivery model of the program

### Step 5: Collating, Summarizing, and Reporting the Data

Both quantitative and qualitative approaches were used to summarize and analyze the extracted data. Descriptive statistics (eg, mean and median) were used to analyze the characteristics of included studies. Descriptive statistics were also used to analyze article and population characteristics for RQ1 and RQ2. A thematic analysis [50] was conducted to categorize the factors from the literature that influence user engagement with digital mental health tools (RQ3) using the sociotechnical framework developed by Sittig and Singh [41]. A thematic analysis was also conducted to characterize the clinical digital health programs that were identified (RQ4). The findings were reported following the PRISMA-ScR (Preferred Reporting Items for Systematic Reviews and Meta-Analyses extension for Scoping Reviews) checklist [51] (Multimedia Appendix 2).

## Results

### Overview

**Figure 1:**
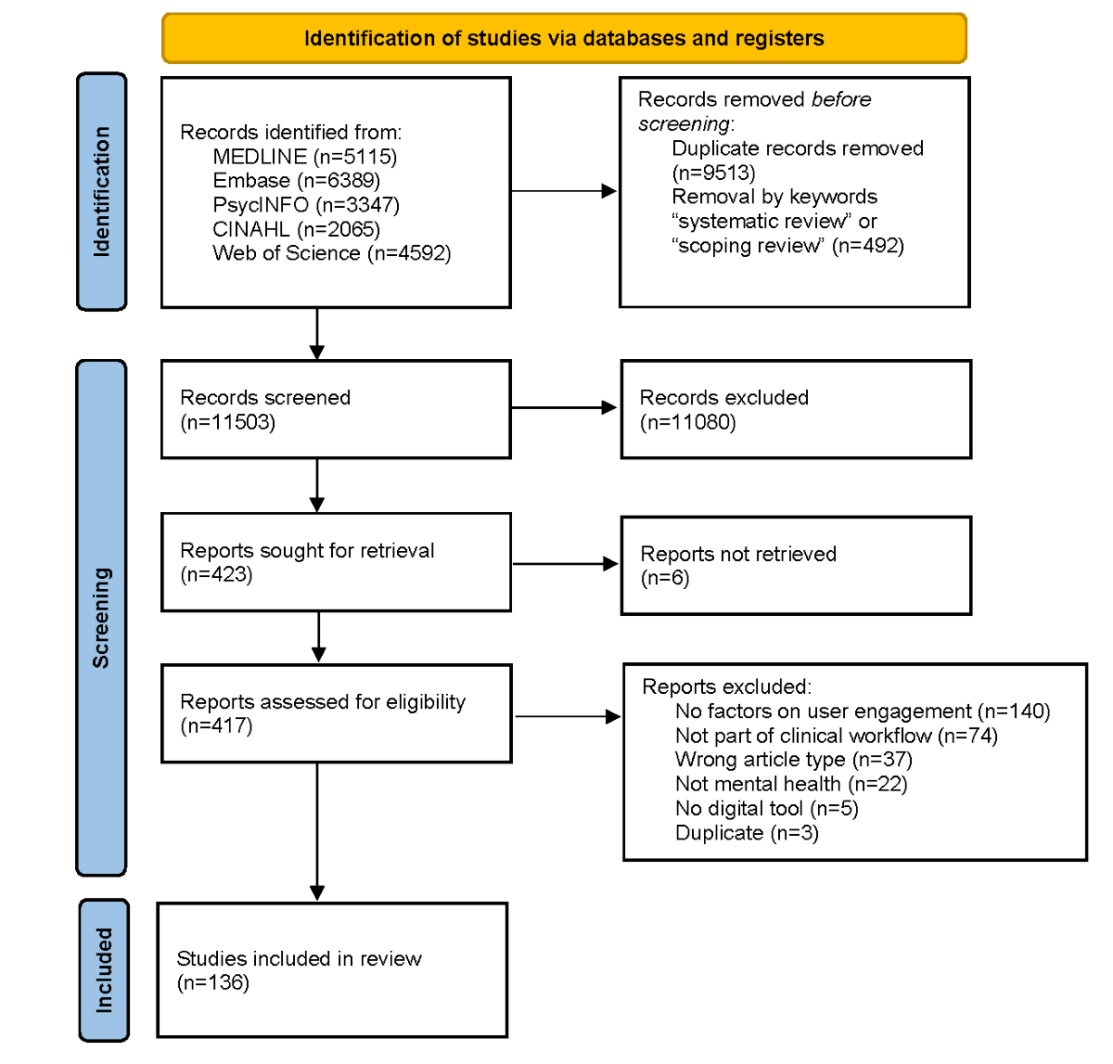
PRISMA-ScR flow diagram.

Study selection results were reported using the PRISMA-ScR diagram [51] (Figure 1). After deduplication, the search strategy yielded 11,503 records for title and abstract screening. Of the 417 articles reviewed for eligibility, 136 (32.6%) were included for analysis. Table 1 summarizes the characteristics of the included articles, and Multimedia Appendix 3 [52-177] provides details of the included articles.

**Table 1:** Characteristics of included articles (n=136).

Characteristics		Articles, n (%)	References
**Year of publication**			
	2006	1 (0.7)	[52]
	2008	1 (0.7)	[53]
	2010	1 (0.7)	[54]
	2011	3 (2.2)	[55-57]
	2012	2 (1.5)	[58,59]
	2013	5 (3.7)	[60-64]
	2014	5 (3.7)	[65-69]
	2015	6 (4.4)	[70-75]
	2016	7 (5.1)	[76-82]
	2017	12 (8.8)	[83-93,177]
	2018	12 (8.8)	[94-105]
	2019	13 (9.6)	[106-118]
	2020	17 (12)	[119,121-136]
	2021	21 (15.4)	[137,138,144-157]
	2022	16 (11.8)	[120,158-162,178-187]
	2023	14 (10.3)	[163-176]
**Country of publication**			
	Australia	11 (8.1)	[54,59,69,110,118,133,165,168,177,178,188]
	Canada	11 (8.1)	[3,73,85,93,116,129,132,148,171,172,174]
	Chile	2 (1.5)	[81,123]
	China	1 (0.7)	[128]
	Denmark	9 (6.6)	[86,101-103,124,141,146,161,175]
	Finland	2 (1.5)	[72,104]
	Germany	7 (5.1)	[122,131,136,154,187,189,190]
	Hungary	1 (0.7)	[56]
	Ireland	2 (1.5)	[121,155]
	Italy	1 (0.7)	[162]
	Netherlands	4 (2.9)	[67,109,111,124]
	Norway	5 (3.7)	[53,63,65,83,173]
	New Zealand	1 (0.7)	[64]
	Spain	5 (3.7)	[90,100,119,163,169]
	Sweden	4 (2.9)	[74,79,80,184]
	Switzerland	1 (0.7)	[70]
	United Kingdom	23 (16.9)	[52,62,66,75,77,89,92,126,127,135,138,142,144,145,147,151,152,160,166,167,176,180,182]
	United States	47 (34.6)	[55,57,58,60,61,68,71,76,78,82,84,87,88,91,94-96,98,105-108,112-114,117, 120,125,130,137,139,140,143,150,153,156-159,164,170,179,181,183,185,186]
**Study design**			
	Case study	6 (4.4)	[102,118,135,150,186,191]
	Cohort study	35 (25.7)	[55,57,59,61,62,68,69,73,78,81,95,96,100,105,112,114,116,117,120,123, 128,130,136,149,152,154,158,159,162,164,169-171,174,177]
	Cross-sectional study	1 (0.7)	[94]
	Feasibility or pilot study	14 (10.3)	[52,56,66,77,84,87,98,99,119,122,131,139,140,157]
	Mixed methods evaluation	11 (8.1)	[75,82,83,115,124,142,143,166,167,175,185]
	Observational study	2 (1.5)	[86,179]
	Qualitative study	43 (31.6)	[53,54,63,65,85,88-90,92,93,101,103,104,107,110,111,113,132-134,137,138, 141,144-146,151,153,155,156,161,163,165,168,172,173,176,178,180,182-184,187]
	Randomized controlled trial	19 (14)	[64,67,70-72,76,79,80,91,97,106,109,121,126,129,147,148,160,181]
	Retrospective study	2 (1.5)	[74,125]
	Usability study	3 (2.2)	[60,108,127]
**Perspective on user engagement (multiselect)**			
	Qualitative	95 (69.9)	[53,54,57,58,60,62,63,65,66,72,73,75,77,79,81-83,85,88-90,92, 93,95,96,100-103,105,107,108,110,111,113,115,116,120-124,126,127,129-134,137, 138,141-149,151-158,160-163,165-173,175-178,180-187]
	Quantitative	55 (40.4)	[52,54,55,59,61,64,66-69,71,72,74-76,80,83,84,91,94-98,106,109, 112,114,116-119,121,122,124-126,128,129,135-137,143,150,159,164,166,167,170,171, 174,175,179,185,186]

In terms of the year of publication, 59.6% (81) of the 136 articles were published within the last 5 years. Publications on this topic originated from 18 countries. The top 4 countries were the United States (n=47, 34.6%), the United Kingdom (n=23, 16.9%), Australia (n=11, 8.1%), and Canada (n=11, 8.1%). A wide variety of study designs were used by the included articles, including cohort studies (n=35, 25.7%), feasibility or pilot studies (n=14, 10.3%), qualitative studies (n=43, 31.6%), and randomized controlled trials (19/136, 14%). With regard to how user engagement was examined, the majority of articles focused on user experience and interest (qualitative; 95/136, 69.9%) as opposed to the degree of use (quantitative; 55/136, 40.4%).

### RQ1: Digital Tools Used in Clinical Care Settings

Of the 136 included articles, 113 (83.1%) disclosed the product or name the tool that was studied (Table 2). Commonly discussed tools in the included articles were FOCUS [60,68,96,113,117] and Apps4Intelligence [116,185], mobile apps for people with schizophrenia; Intellicare, a platform that focuses on navigation and recommendation of digital tools [84,106]; and Horyzons, a social therapy platform for first-episode psychosis [133,134]. In terms of tool type, the majority were websites (62/136, 45.6%) and mobile apps (49/136, 36%). Notably, 6 (10%) of the 62 websites were patient portals from health care organizations [132,158,159,171,173]. From a client-facing perspective, these digital tools offered a wide range of functionalities (Table 2). Specifically, among computerized interventions, resources, and applications (111/136, 81.6%), some examples include tools that were focused on delivering cognitive behavioral therapy [57,59,63-65,74,79,80,89,91,98,102,111,128,184] and educational workbooks or resources [54,56,66,123,125] for patients to complete. Regarding clinician involvement in the support and use of the tool, most provided support in a coaching (49/136, 36%) or comprehensive (53/136, 39%) manner [4]; for example, for the FOCUS app and Intellicare platform, clinicians would guide users on the use of the tool and develop a plan around how the tool should be used throughout the care journey [84,106,117]. Other tools, such as the BRAVE platform, required clinicians to use it in a more comprehensive manner to review results and to discuss and plan next steps in care through and with the platform [118].

**Table 2:** Characteristics of digital tools examined in the included articles (n=136).

Characteristics		Articles, n (%)	References
**Type of digital health tool (multiselect)**			
	Chat group	5 (3.7)	[56,78,91,139,148]
	Computer software	2 (1.5)	[75,187]
	Mobile app	49 (36)	[60,62,68,84,88,92,93,95,96,101,103,105-107,109,113,116,117,119,120,126, 131,135,137,138,140,142,145,146,152,153,156,157,160-164,166,167,169,170,175,176, 179-182,185,186]
	SMS text messaging or texting	12 (8.8)	[52,53,57,67,69,70,72,77,87,108,147,150]
	Website	62 (45.6)	[54,56,58,59,61,63-66,71,73,74,79-83,86,89,90,94,97-100,102,104, 110-112,114,115,118,121-125,127-130,132-134,136,139,141,143,144,149,151,154,155, 158,159,168,171,173,177,183,184]
	Telephone software	2 (1.5)	[76,172]
	Wearable and virtual reality	11 (8.1)	[85,162,165-167,169,170,174,176,178,181]
**Functionality of digital health tool (multiselect)**			
	Big data	2 (1.5)	[75,161]
	Computerized interventions, resources, and applications	111 (81.6)	[54,56,57,59-70,74,75,77,79-84,86-90,92-94,96-118,121-136,138-146, 148-160,162-164,166,168-171,173,175,176,178,179,181-186]
	Peer support	11 (8.1)	[56,58,83,93,107,122,133,139,148,149,185]
	Telehealth and telemedicine	54 (39.7)	[52,53,55,57,64,67,68,70-73,75,76,78,81,83,88,91,94-96,98,105, 107,113,116,117,119-123,132,134,137,146,147,150,153,156-159,162,166,167,169,170, 172,176,177,180,183,185,186]
	Virtual reality	3 (2.2)	[141,165,187]
	Wearable computing and monitoring	9 (6.6)	[85,92,116,119,139,166,174,178,181]
**Level of clinician involvement**			
	Promotion	7 (5.1)	[52,59,72,86,87,125,139]
	Case management	5 (3.7)	[73,75,107,134,148]
	Coaching	49 (36)	[57,61,64,65,67-71,74,79-82,84,89,90,97,99,100,104-106,109,110,112, 113,115,116,126,128-130,138,140-146,149-151,153,155-157,163]
	Symptom focused	22 (16.1)	[54,63,66,91,92,94,95,101-103,108,121,122,124,131,133,136,137,147,152,159, 164,177,182,184]
	Comprehensive	53 (39)	[53,55,56,58,60,62,76-78,83,85,88,93,96,98,111,114,117-120,123,127, 132,135,138,154,158,160-162,165-176,178-181,183,185-187,192]

### RQ2: Characteristics of Populations Using Digital Mental Health Tools in Clinical Care Settings

With regard to the population of end users (Table 3), the majority of articles (101/136, 74.3%) examined tools that were developed for the adult population. Only a few articles (12/136, 8.8%) had a focus on younger adults (aged <18 years), while the study by Sheeran et al [55] evaluated a depression management program for individuals aged >65 years receiving home care services. Moreover, the target conditions of the tools varied significantly, with a little more than one-third of the articles (47/136, 34.6%) examining tools that supported >1 type of mental health condition. Regarding condition-specific tools, the most frequent conditions examined were depression (26/136, 19.1%) and schizophrenia spectrum and related psychotic disorders (20/136, 14.7%). Finally, regarding the duration of participation, 52.9% (72/136) of the studies reported how long participants were asked to use the tool. Of these 72 studies, 42 (58%) asked participants to use the tool for <3 months.

**Table 3:** Characteristics of populations (patients) examined in the included articles (n=136).

Characteristics		Articles, n (%)	References
**Age range (years)**			
	<18	12 (8.8)	[77,99,104,107,118,120,133,139,144,149,151]
	≥18	101 (74.3)	[53-55,57,58,60-64,66-69,72-76,78-87,90-98,100,105,106,108-117,119, 121-123,125-128,130,134-138,140-143,146,147,150,152,153,155,157-166,169,171-176, 178-187,192]
	Mixed	12 (8.8)	[52,56,59,101,103,129,138,145,148,167,168,170,177]
	Not reported	11 (8.1)	[65,70,71,88,89,102,124,131,132,154,156,158]
**Mental health condition**			
	Anxiety disorders	11 (8.1)	[64,74,98-100,118,128,130,141,144,161]
	Bipolar and related disorders	7 (5.1)	[54,76,78,92,121,157,174]
	Depressive disorders	26 (19.1)	[55,57,61,63,65-67,81,85,86,97,109,111,112,115,125,127,131,138,142,149, 153,154,163,176,179,183]
	Feeding and eating disorders	7 (5.1)	[52,56,58,80,82,91,101]
	General and undifferentiated mental illness	47 (34.6)	[59,60,69,73,75,77,79,83,84,90,93,94,96,102,104-106,114,119,120,122, 124,129,132,134,135,137,140,147,148,150,155,158,159,164,166,168,170,171,173,177, 178,180,181,184,186,187]
	Neurodevelopmental disorders	3 (2.2)	[71,139,151]
	Obsessive-compulsive and related disorders	2 (1.5)	[89,165]
	Personality disorders	1 (0.7)	[182]
	Schizophrenia spectrum and related disorders	20 (14.7)	[60,62,68,72,87,96,103,108,116,117,126,133,138,143,145,146,152,162,169,185]
	Substance-related and addictive disorders	6 (4.4)	[70,95,107,110,172,175]
	Trauma and stress-related disorders	4 (2.9)	[88,123,136,160]
**Duration of participation**			
	<6 wk	12 (8.8)	[22,55,62,86,87,120,142,158,162,164,185,186]
	6 wk to 3 mo	30 (22.6)	[22,57,78,82,83,85,97,99,100,112,113,119,121,123,128,131,138,140,144,148-150, 152,160,161,163,167,169,170,184]
	4 mo to 1 y	27 (19.9)	[52-54,56,61,64,66,69,70,73,76,77,80,92,95,98,101,117,126,143,146, 147,166,171,174,181,182]
	>1 y	3 (2.2)	[74,133,176]
	Not applicable or not reported	64 (47.1)	[58-60,63,65,67,68,71,72,75,79,81,84,88-94,96,102-112,114-116,118, 122,124,125,127-132,134-139,141,145,151,153,154,156-159,165,168,172,173,175, 177-180,183,187]

### RQ3: Factors That Influence User Engagement With Digital Mental Health Tools

#### Overview

Among the included articles, 26 factors were identified across 7 (88%) of the 8 categories outlined in the sociotechnical framework developed by Sittig and Singh [41]. The categories with the most number of factors were human-computer interface (5/26, 19%) and people (5/26, 19%). No factors were coded in the system measurement and monitoring component of the framework because this was considered an outcome of user engagement (eg, user analytics) [41]. Table 4 presents an overview of the factors and their descriptions; further details are provided in the subsections that follow.

**Table 4:** Overview of the factors related to user engagement with digital mental health tools in clinical care settings.

Factors			Descriptions		References
**Hardware and software computing infrastructure**					
	Modality of the tool	Ability to effectively use the tool across multiplatform systems to ensure compatibility with end-user devices		[53,60,63,67,70,76,85,88,90,98,110,120,133,137,138,141-143,147,148, 168,169,176,180]	
	Access to the tool	Ease of access to the technology outside of the clinical setting		[58,71,88,111,132,134,143,150,153,167,168,172,180]	
	Technical challenges	Technical glitches and issues affecting the full use of the tool		[55,56,75,81,122,129,131,138,144,169,176]	
**Clinical content**					
	Personalization of the content	Tailored delivery of questions and feedback based on the preferences and current status of the individual		[63,64,78,81-83,92,93,103,105,108,113,123,127,131,135,140,142,144,145, 147,149,155,157,158,163,177,178,184]	
	Delivery of the content	Delivery of content using the right language, length, medium, and tone		[60,63,82,100,111,115,124,127,130,138,142,146,158,168,183,184]	
	Spans across the patient journey	Tools that adapt to the evolving needs of the individual across the care journey		[54,110,133,142,147,152,158,177]	
	Appropriate follow-up and user interaction	Response and feedback are actionable, trauma informed, and informed by lived experiences		[82,92,103,108,113,129,130,132,134,138,145,152,153,156]	
**Human-computer interface**					
	Preferred modality of content delivery	Availability and alignment of content delivery to end-user preferences (eg, audiobook or video)		[53,55,57,60,62,71,75,82,90,92,96,105,108,132,138,140,141,144,146,155,184,187]	
	Desired interaction duration	Expected duration and resources needed to use the tool		[64,75,90,108,110,115,118,119,121,150,162,167,168,170,182]	
	Usability	How easy or difficult it is to complete a task		[82,89,92,103,107,110,111,116,131,132,134,138-140,143,152,162,163,167,175-178,181,183]	
	Feedback and incentivization	Providing immediate feedback and rewards for responses and routine use of the tool		[52,57,58,70,85,92,101,103,127,135,142,145,147,157,158,161,162,178,180,186]	
	Interoperability with other platforms	Bidirectional connectivity to other platforms and tools (eg, a calendar app)		[73,154]	
**People**					
	Affordability	Costs to use the tool		[56,110,111,132,142,145,153,167,168]	
	Presence of clinician support	Active support from clinicians to encourage use of the tool		[56,66,75,88,111,115,123,126,135,145,150,151,153,158,160,161,163,165, 168,171,175,176,180-182,184,187]	
	Presence of support from family and friends	Active support from family and friends to encourage use of the tool		[56,66,82,83,85,104,110,121,139,140,150,155,156,181]	
	Clinical condition and critical events	The impact of the mental health condition and related events (eg, suicide attempt or loss of home)		[53,54,59,63,65,69,71,73-75,80-82,85-87,92,93,100,101, 103-106,108,114,116,119,120,122,123,127,129-133,135,136,145,146,148, 149,153,155,156,158,162,165,167,168,170,174,176,179,182-187]	
	Sociodemographic characteristics	Impact related to the characteristics of the population such as location, ethnicity, and age		[54,57,76,84,91,94,97,112,117,128,136,148,153,154,159,164,166,168,174,179]	
**Workflow and communication**					
	Incorporation into care workflows	Embeddedness of the tool into the daily practice and processes of clinical care		[53,54,63,78,82,88,89,92,93,96,99,101-105,107,108,111,115,120, 122,124,127,131,133,134,137,138,157,158,161,162,167,168,170,177,181,184]	
	Expectation setting	Expectations for tool use as agreed upon or imposed by patients, families, and clinicians		[53,63,65,67,68,74-77,79,82,83,89,103,105,108,110,111,113,120, 124,129,135,137,138,144,145,147,149,153,156,158,160,165,173,175,178,180]	
	Delivery of education	Guidance on use and interpreting data on the platform		[83,139,143]	
	Support for use	Providing peer and technical support to help end users identify opportunities for meaningful use of the tool		[54,63,71,97,100,109,110,116,118,128,129,131,132,142,151,152,154-156,171,176,183]	
**Internal organizational policies, procedures, and culture**					
	Privacy and security	Safeguards, regulations, and policies in place to support privacy and security requirements from end users		[82,96,105,126,134,135,145,152,172]	
	Management buy-in	Endorsement and support to build capacity and change management for digital health from clinical and health system leaders		[77,104,176]	
	Administrative burden on clinicians	The administrative burden on clinicians when working with patients and families on the platform		[75,83,95,111,115,121,127,134,137,142,158,165,168,175]	
**External rules, regulations, and pressures**					
	Guidelines for use	Establishment of expectations for tool use by patients, families, and clinicians (eg, liability)		[83,89,95,101,102,111,158,161,165,172]	
	Health system infrastructure and reimbursement	Endorsement and capacity building for patients, families, and clinicians to use the tool (eg, billing)		[66,82,83,89,102,137,151,158,161,172,173,176]	

#### Hardware and Software Computing Infrastructure

Within the hardware and software computing infrastructure category, three factors were identified: (1) modality of the tool, (2) access to the tool, and (3) technical challenges. Of the 136 included articles, 25 (18.4%) highlighted how the tool’s modality (eg, mobile app or website) influenced engagement; for example, participants in Big White Wall (now Togetherall), a peer support network for mental health service users, found that the lack of a mobile app was problematic for easy access to the platform when needed [148]. Others also experienced issues when trying to run the platform on different or older models of mobile devices (eg, Android or iPhone) [137,144]. Moreover, 13 (9.6%) of the 136 articles outlined the importance of considering how patients would be able to access the tool. Medalia et al [143] examined the feasibility and acceptability of a cognitive remediation platform, which allows patients with schizophrenia to complete some of their homework remotely in public settings or at home. While the intent of the platform was to reduce the need for attending a clinic multiple times per week, the authors found that system and bandwidth requirements made it difficult for individuals to use the platform effectively in public settings (eg, libraries). Finally, 11 (8.1%) of the 136 articles reported challenges related to bugs and limitations, which influenced the ability of patients to stay engaged, particularly in platforms still in early development [81,146].

#### Clinical Content

There were four factors related to how the clinical content on the platform influenced user engagement: (1) personalization of the content, (2) delivery of the content, (3) spans across the patient journey, and (4) appropriate follow-up and user interaction. For content personalization, 29 (21.3%) of the 136 articles highlighted the importance of tailoring the content to users’ needs and preferences to enhance engagement; for example, in remote mood tracking tools, it was identified that repeatedly using the same wording for questions often felt impersonal and repetitive, regardless of whether a user’s condition improved or deteriorated [81,145]. This issue also extended to the modules [138], educational materials and treatment [131,188], and messages delivered within the therapeutic components [144,188]. In addition, 16 (11.7%) of the 136 articles highlighted the need to consider how the content is delivered; for example, in the case of clinical notes within patient portals, there is a need to ensure patient-centric wording in condition descriptions to ensure that the documentation supports patient care [108,158] and aligns with a patient-centric approach [111,131,146].

Furthermore, a few articles (8/136, 5.9%) looked at the importance of considering each patient’s treatment stage. As needs and acuity levels differ from admission to discharge, use cases and needs related to engagement will likely vary [193]; for example, participants using the Mindframe platform highlighted how it enabled them to understand how their care has evolved since they began treatment [103]. Valentine et al [133] also reported that patients at the beginning of treatment are less likely to transparently report their mood due to concerns about increasing the intensity of their care. Thus, adjusting the content and its delivery may be influenced by the patient journey. This also extends to the need for appropriate follow-up and user interaction such that the tool can generate bidirectional conversations between the tool and the patient (15/136, 11%). While providing feedback and reminders could be useful in promoting engagement over time, some participants reported feeling scrutinized and judged based on the responses they received when they were unable to complete an assigned task [138].

#### Human-Computer Interface

Five factors related to the human-computer interface of the platform were identified: (1) preferred modality of content delivery, (2) desired interaction duration, (3) usability, (4) feedback and incentivization, and (5) interoperability with other platforms. Building on the need to have various modalities for content delivery, 22 (16.2%) of the 136 articles outlined the need to allow patients to choose their preferred modality for receiving clinical content [110]; for example, a number of therapists in the study by Rodda et al [110] mentioned that while patient preferences can vary significantly, there is typically a strong preference for a specific modality. This also extends to the expected duration of patient interaction with the platform (15/136, 11%). In the same study, therapists found that overly long content and modules can be overwhelming, making it difficult for patients to complete them and stay engaged over time [110].

Other key areas identified in the included articles were usability and interoperability with other tools. In particular, ease of use, navigation, and accessibility within the tool’s features were reported to be important contributors to user engagement (26/136, 19.1%). The lack of an easy-to-use interface can be detrimental to the utility and use of the tool; for example, in the addiction comprehensive health enhancement support system examined by Hussey and Flynn [107], the emergency call feature was placed in a prominent location for users to access when needed. However, because of its placement, it would be clicked accidentally even when help was not required.

Finally, with the growth of gamification, 20 (14.7%) of the 136 articles looked at the potential benefits and risks of providing immediate feedback and incentives to enhance engagement. While Lindgreen et al [101] found that the delivery of feedback alongside reminders and nudges was considered useful in enhancing engagement, it was important to be considerate and trauma informed; for example, in their study on an eating disorder app, it was observed that simply sending reminders to eat could be perceived as condescending and hence detrimental if not delivered appropriately. Some of these reminders and enhancements should also be interoperable with other platforms that individuals currently use on their device (eg, a calendar app; 2/136, 1.5%).

#### People

Several factors related to patients as end users were also identified as influencing user engagement. These included (1) affordability, (2) sociodemographic characteristics, (3) presence of clinician support, (4) presence of support from family and friends, and (5) clinical condition and critical events.

With regard to affordability and sociodemographic characteristics, a few articles (9/136, 6.7%) discussed the challenges related to having devices for use with the digital mental health tool. While studies frequently provided a digital device for use with the digital mental health tool, many individuals highlighted that once device and data access were no longer provided, engagement decreased drastically [95]. Other studies using quantitative data highlighted how sociodemographic characteristics such as age and ethnicity (21/136, 15.4%) have also been found to be related to the extent of use [128,189]. Thus, understanding how sociodemographic characteristics and affordability intersect with engagement for a particular population is critical.

The presence of clinician support as well as support from family and friends was another key factor influencing engagement. Of the 136 articles, 27 (19.9%) spoke about the importance of having a clinician support and be engaged throughout the use of the digital mental health tool; for instance, participants in the study on 3 stepped care tools by March et al [118] highlighted the role of clinicians in the customization and tailoring of strategies and implementation approaches to ensure that the tool aligns with the needs of end users. In the event that nonadherence was observed, there was an opportunity to intervene and provide adequate support. Moreover, the presence of support from family members and friends was reported to enhance engagement by offering encouragement, time, and space for patients to use the tool (14/136, 10.2%). Nitsch et al [82] also shared that participants found it meaningful to do it (engage in the tool) for their family and friends. Finally, 61 (44.9%) of the 136 studies discussed the role of patients’ clinical condition and critical events; for example, some individuals receiving care for general anxiety disorder reported feeling too anxious to use a digital tool [64], whereas other studies found that those with anxiety had higher levels of engagement than individuals with depression [74]. In addition, some clinicians highlighted that individuals with psychosis and active suicidal ideations may not be suitable candidates with regard to relying on a digital system for support [153].

#### Workflow and Communication

Several factors related to workflow and communication were identified. One key aspect outlined by several articles (40/136, 29.4%) was the tool’s ability to be incorporated into clinical care delivery. This can take various forms, such as enabling working together on worksheets through the platform [127] or providing advance information before a clinical visit [158]. In the case of a monitoring app for eating disorder, the lack of discussion regarding the information provided by clients during each session led to discouragement and a loss of trust in the clinician [101]. Thus, there is a need to set clear expectations (39/136, 28.7%) with clients about the appropriate and effective use of the tool as part of clinical care settings. Some individuals used the platform as a means to seek support after hours [113], while others held perceptions that the tool would take over the therapist’s role [151].

Other factors within this category included the need to deliver education and support to patients (3/136, 2.2%), as well as provide adequate support throughout the duration of tool use to encourage engagement (22/136, 16.2%). The study by Morrison et al [66] highlighted the importance of having a care manager to provide guidance on the appropriate and meaningful use of the tool in the overall care.

#### Internal Organizational Policies, Procedures, and Culture

An emerging number of studies have highlighted the impact of internal organizational policies, procedures, and culture on user engagement. Foremost, privacy and security are considered pinnacle issues for patients (9/136, 6.7%). As these tools often collect, use, and analyze personal health information, patients have expressed concerns about how their data are collected and whether the data are safe and secure [82]. Moreover, some clients have queried whether the data would be anonymized or whether access would be limited to their care team [105]. Thus, clearly communicating and outlining privacy and security considerations with regard to the tool would be important for fostering engagement and encouraging users to enter private, sensitive information over time.

In addition, buy-in and expectations from organizational administration and leadership influenced user engagement (3/136, 2.2%). Kurki et al [104] discussed how the expectations and accountability placed on clinicians affected their ability to encourage clients to engage with the tool as part of clinical care. This is particularly important when the app includes content related to suicide and self-harm because it can impact how clinicians should be responding to these incidents in a timely manner [95,107]. Administrative burden was raised as another issue for clinicians (15/136, 11.0%). While these studies outlined the administrative burden related to clients completing repetitive surveys [119], the workload for clinicians was cited as a critical barrier to reviewing and making meaningful use of the data for care [121]. Without protected time and adequate alignment and expectations regarding the time and effort required to use the tool, the workload often became a barrier to continued engagement over time.

#### External Rules, Regulations, and Pressures

Two factors related to the external rules, regulations, and pressures of the health system were identified. The first involves guidelines for tool use as prescribed by regulatory bodies and professional colleges (10/136, 7.3%). Folker et al [102] discussed the need to push system-level policies that support the uptake and adoption of digital mental health tools such that there is overall encouragement and support for the use of these emerging approaches in care. The other factor, highlighted by 12 (8.8%) of the 136 articles, is the financial reimbursement of clinicians for using these tools [115]. Given the administrative burden associated with the use of these tools, there is a strong need for clinicians to be reimbursed for the time spent in using these tools for care. It may also be useful to account for this workload within clinicians’ daily responsibilities.

### RQ4: Clinical Programs That Embed Digital Mental Health Tools

Some of the studies (20/136, 14.8%) provided a brief description of how digital mental health tools can be embedded within clinical programs [53,55,66,75,104,109,113,120,121,129, 143,146,154,155,160,163,168,177,178,182]; for example, Kemmeren et al [109] outlined a clinical workflow specifying when these tools should be introduced and used with patients, as well as the intended duration and use of the various modules within the blended cognitive behavioral therapy tool. In addition, these articles described training for clinicians on using the tool with patients and provided guidance on how to navigate and support patients in its use [55,104]. For the FOCUS app [113], information was provided on the frequency and approach for checking in with clients regarding the use of the digital tool.

## Discussion

### Principal Findings

To our knowledge, this is one of the first reviews to focus on identifying a preliminary, comprehensive framework of factors that influence user engagement with digital mental health tools in clinical care settings [24]. In this review, 26 factors were identified from 136 articles that spanned across the components of the sociotechnical framework developed by Sittig and Singh [41]. These factors illustrate how technology, relevant stakeholders, and environment can influence user engagement with digital mental health tools. Moreover, based on the studies that examined the clinical programs that embed digital mental health tools, there is increasing discussion on how these tools can be delivered in real-world environments.

As there are a growing number of studies looking at ways to address the ongoing challenges of user engagement [194-196], this work has contributed to a better understanding of the dynamics of, and contributors to, effective user engagement in a clinical environment across individual, organizational, and health system levels [37]. Given that the focus of this scoping review was on digital mental health tools in clinical care environments, this work will help inform the development of implementation approaches and strategies for ensuring effective engagement and integration of digital mental health tools in clinical care settings and models of care. In addition, as digital tools continue to proliferate in the clinical environment, engagement with these tools has become an extension of how patients engage with clinicians, receive care, and navigate the clinical environment [24,33,197]. Hence, engagement with digital tools can be a key factor in the overall success of treatment. The specific impact of this work on several key areas of user engagement is discussed in the following paragraphs.

First, as outlined in the Introduction section, a number of existing frameworks have explored the factors that influence user engagement with digital tools [27,32]. In this work, while many of the identified factors are consistent with the findings of previous reviews, the application of the sociotechnical framework developed by Sittig and Singh [41] was useful in 2 ways. Given the overarching goal of developing interventions to enhance effective user engagement, the sociotechnical framework helped to further break down the factors related to user engagement by focusing on digital mental health tools in clinical care settings; for example, technology-related factors were further characterized into factors that pertain to the content, the technology itself, and the human-computer interface [41]. Second, rather than providing a “laundry list” of factors within the categories, the sociotechnical framework helped to clarify and conceptualize the interplay of factors across its components. Thus, this work extends the broad scope by providing more specificity on how some of the factors would interact with each other through a sociotechnical lens. However, there were also challenges in applying the sociotechnical framework. Given that many of the factors are closely related, it was difficult to articulate the interconnectedness and interactions across the various components of the framework. Future work should further explore these nuances, including how these factors are connected and how factors specific to different end users (eg, patients and clinicians) interact.

The findings from this work also provide more insights into factors specific to the discipline and the implementation setting of these tools. Borghouts et al [32] conducted a similar scoping review but focused more broadly on digital mental health tools and identified 16 factors that span across the user, program, technology, and environment. While many of these factors align with those in this review, the relatively narrower focus of our review uncovered 2 additional facets specific to tools integrated within the clinical environment. First, the importance of support from clinicians was identified as a critical component. While many studies have looked at how clinician-guided approaches can help improve engagement with digital mental health tools [198], there remains a lack of guidance and best practices on how best to support patients throughout their care journey [138]. As such, it may be useful to develop best practices that is informed by a customer experience approach through user experience perspectives [199].

Moreover, as the tool will be embedded in the health care organization, it is critical to consider the workflows and internal processes. Establishing clear guidelines on the accountability and role of each member of the circle of care in the use of digital tools will ensure that end users understand what constitutes appropriate, safe, and effective use [20]; for example, for tools that are not actively monitored after hours, it is critical to reiterate through policy that patients and clinicians understand that the tool is not to be used during emergency crises [108]. Another important aspect of workflow and organizational policies is ensuring protected time for clinicians to use digital tools with patients. In the Canadian context, where physicians rely on a fee-for-service model, there is a need to establish remuneration policies so that clinicians can be compensated for the time and administrative burden associated with the use of these tools [115]. With clinician burnout related to digital tool use becoming a tremendous challenge in the last few years [200-204], there is a need to consider the impact of these tools on documentation and administrative burden. Thus, the findings from this review highlight the need to address engagement not only at the patient level but also at the clinician and system levels. In particular, investing in factors that support clinicians and the broader health system is equally important to ensure that all roles and key players can engage with the tool in a meaningful way, which can potentially be more cost-effective than focusing solely on patient-level factors [198,205,206]. Future work should capture how various organizations address the factors at these levels.

Finally, the findings from this work have emerging implications for mental health clinicians, administrators, developers, and researchers. It is also important to note that while these factors were developed from mental health literature, it is likely that many of these factors are also relevant for digital health in other clinical areas [197,198]. For clinicians, it would be useful to consider these factors in formulating a plan and encouraging patients in the use of digital mental health tools in clinical care delivery [207]. Developers can consider these factors throughout the software development lifecycle and consider how these factors should be integrated into the features and development (eg, push reminders) of digital mental health tools. Educators and administrators may consider leveraging these factors to develop appropriate guidelines and curricula to enhance the capacity and readiness of current and future health care professionals to use these tools in their practice [208]. Similarly, administrators should consider these findings in developing a conducive environment for enhancing engagement with digital health tools in clinical practice. Finally, researchers can build on these findings to develop guidance (eg, tool kits) for designing tools keeping user engagement in mind. In addition, interventional studies can examine the impact of these factors on user engagement with digital mental health tools in clinical care settings.

### Limitations

Several limitations should be considered when interpreting and applying the preliminary framework of factors from this literature review. First, given the nature of scoping reviews, the quality of the included articles was not assessed, and the literature search is not assumed to be exhaustive [46,51]. Second, this work primarily focused broadly on the patient population and, given the diverse characteristics of the populations included in this review, it was difficult to identify nuances and differences in impact across various sociodemographic and clinical populations.

In addition, no studies were identified that looked at user engagement with digital mental health tools from the perspectives of caregivers and family members, despite their importance in mental health care [209]. As studies such as that by Simões de Almeida [210] have shown, exploring the prominence of various factors among different populations may be of interest. Finally, the preliminary framework of factors was developed based on the academic literature and were not validated with subject matter experts. This will be addressed in a subsequent study.

### Future Directions

Several future directions have been identified to further strengthen and validate the framework of factors for digital mental health tools in clinical care settings. First, given the large number of factors identified from the included articles, there is a strong need to validate and identify the impact of these factors on user engagement. A Delphi study will be conducted to validate and identify any factors not found in this literature review [211]. Second, as the majority of the included studies (49/136, 36.1%) focused on the use of mobile apps, there was limited insight into the use of emerging tools such as patient portals and wearables. Finally, it would be imperative to explore how these factors influence user engagement in different clinical contexts.

### Conclusions

A total of 26 factors influencing user engagement with digital mental health tools within clinical care settings were identified from the academic literature. These factors spanned across the categories of the sociotechnical framework, highlighting the complexity and interconnectedness of the factors in influencing user engagement. Future work should focus on identifying the factors considered essential for influencing user engagement and conducting exploratory studies to examine the differential impact of these factors on user engagement.

## Appendix

Multimedia Appendix 1 Search strategy.

Multimedia Appendix 2 PRISMA-ScR (Preferred Reporting Items for Systematic Reviews and Meta-Analyses extension for Scoping Reviews) checklist.

Multimedia Appendix 3 Details of the included articles.

## Abbreviations

MeSH: Medical Subject Headings
PRISMA-ScR: Preferred Reporting Items for Systematic Reviews and Meta-Analyses extension for Scoping Reviews
RQ: research question

## Data Availability

All data generated or analyzed during this study are included in this published paper and Multimedia Appendix 3.
