# Supplementary material for: Application of a Sociotechnical Framework to Uncover Factors That Influence Effective User Engagement With Digital Mental Health Tools in Clinical Care Contexts: Scoping Review
Source: J Med Internet Res. 2025 Apr 28;27:e67820. doi: 10.2196/67820 (PMC12070020; doi:10.2196/67820)
Supplement: Multimedia Appendix 1 [file jmir_v27i1e67820_app1.pdf]

MEDLINE

Database: Ovid MEDLINE: Epub Ahead of Print, In-Process & Other Non-Indexed Citations, Ovid MEDLINE® Daily and Ovid MEDLINE® <1946-Present>
Search Strategy:
----
1 mental disorders/ (169912)
2 mental health/ (47556)
3 mentally ill persons/ (6342)
4 mental health services/ (36232)
5 (mental$ ill$ or mental$ disorder$ or mental$ health$).mp. (397690)
6 (psychiatric adj3 (client$ or patient$ or inpatient$ or service$ or treat$ or program$)).mp. (51467)
7 or/1-6 [Mental Health Set] (420908)
8 Suicidal Ideation/ or Suicide/ or suicid$.mp. (103092)
9 Hallucinations/ or Hallucinat$.mp. (19860)
10 8 or 9 [Mental Health Symptoms Set] (122272)
11 Crisis Intervention/ or crisis interven$.mp. (6679)
12 Psychotherapy/ or psychotherap$.mp. (94808)
13 Cognitive Behavioral Therap$.mp. or Cognitive Behavioral Therapy/ or (cognitive adj2 therap$).mp. or behave$ therap$.mp. (38497)
14 11 or 12 or 13 [Mental Health Treatment Set] (130304)
15 7 or 10 or 14 [Total Set for Mental Health] (610684)
16 Mobile Application$/ (8758)
17 (mobile adj2 (app$ or therap$)).mp. (15004)
18 (mhealth or m-health).mp. (7162)
19 wearable$.mp. (18685)
20 patient$ portal$.mp. or Patient Portals/ (1633)
21 text messag$.mp. or Text Messaging/ (6646)
22 (mobile or technology-assisted or computer-based or internet-based or information technology or web-based or technology-mediated or technology-enabled).mp. (188404)
23 virtual realit$.mp. or Virtual Reality/ (13718)
24 telemedicine/ or (teletherap$ or telepsychiatr$ or telemed$ or telehealth or tele-therap$ or tele-psychiatr$ or tele-med$ or tele-health).mp. or telemental$.mp. or teletherap$.mp. (47957)
25 video game$.mp. or Video Games/ or digital tool$.mp. (9028)
26 16 or 17 or 18 or 19 or 20 or 21 or 22 or 23 or 24 or 25 [Digital Health Tools set] (267979)
27 (dropout$ or drop out$).mp. (26257)
28 adheren$.mp. (209551)
29 ((user$ or patient$ or caregiver$ or care giver$) adj2 (engag$ or experience$)).mp. (127268)
30 uptak$.mp. (415368)
31 accept$.mp. (549841)
32 usability.mp. (16288)
33 27 or 28 or 29 or 30 or 31 or 32 [User Engagement Set] (1297563)
34 15 and 26 and 33 [Total Set] (3409)
35 34 not (exp animals/ not humans.sh.) (3408)

****

EMBASE

Database: Embase Classic+Embase
Search Strategy:
----
1 mental disease/ (264382)
2 mental health/ (165267)
3 mental patient/ (30552)
4 mental health service/ (63233)
5 (mental$ ill$ or mental$ disorder$ or mental$ health$).mp. (432231)
6 (psychiatric adj3 (client$ or patient$ or inpatient$ or service$ or treat$ or program$)).mp. (75523)
7 1 or 2 or 3 or 4 or 5 or 6 (642448)
8 suicide/ or suicidal ideation/ or suicidal behavior/ (95207)
9 suicid$.mp. (148800)
10 hallucination/ (29118)
11 Hallucinat$.mp. (47329)
12 8 or 9 or 10 or 11 (193366)
13 crisis intervention/ (6733)
14 crisis interven$.mp. (7576)
15 psychotherapy/ (102462)
16 psychotherap$.mp. (129625)
17 Cognitive Behavioral Therap$.mp. or Cognitive Behavioral Therapy/ or (cognitive adj2 therap$).mp. or behave$ therap$.mp. (68508)
18 13 or 14 or 15 or 16 or 17 (188248)
19 7 or 12 or 18 [Total set for mental health] (919420)
20 mobile application/ (15802)
21 (mobile adj2 (app$ or therap$)).mp. (21685)
22 (mhealth or m-health).mp. (6940)
23 wearable$.mp. (20165)
24 patient$ portal$.mp. (2113)
25 text messaging/ (6128)
26 text messag$.mp. (9188)
27 (mobile or technology-assisted or computer-based or internet-based or information technology or web-based or technology-mediated or technology-enabled).mp. (270355)
28 virtual reality/ (20568)
29 virtual realit$.mp. (26026)
30 telemedicine/ (33762)
31 (teletherap$ or telepsychiatr$ or telemed$ or telehealth or tele-therap$ or tele-psychiatr$ or tele-med$ or tele-health or telemental$ or teletherap$).mp. (57989)
32 video game/ (4543)
33 (video game$ or digital tool$).mp. (8827)
34 20 or 21 or 22 or 23 or 24 or 25 or 26 or 27 or 28 or 29 or 30 or 31 or 32 or 33 (371602)
35 (dropout$ or drop out$).mp. (29541)
36 adheren$.mp. (286571)
37 ((user$ or patient$ or caregiver$ or care giver$) adj2 (engag$ or experience$)).mp. (207934)
38 uptak$.mp. (581114)
39 accept$.mp. (686151)
40 usability.mp. (20512)
41 35 or 36 or 37 or 38 or 39 or 40 (1750037)
42 19 and 34 and 41 (4274)

****

PSYCINFO

Database: APA PsycInfo <1806 to October Week 2 2021>
Search Strategy:
----
1 Mental Disorders/ (89444)
2 Mental Health/ (74022)
3 Psychiatric Patients/ (28986)
4 Mental Health Services/ (36260)
5 (mental$ ill$ or mental$ disorder$ or mental$ health$).mp. (390969)
6 (psychiatric adj3 (client$ or patient$ or inpatient$ or service$ or treat$ or program$)).mp. (73183)
7 1 or 2 or 3 or 4 or 5 or 6 (427637)
8 Suicidal Ideation/ (10321)
9 Suicide/ (28689)
10 suicid$.mp. (74722)
11 Hallucinations/ (3620)
12 Hallucinat$.mp. (18052)
13 8 or 9 or 10 or 11 or 12 (92007)
14 Crisis Intervention/ (3843)
15 crisis interven$.mp. (8365)
16 Psychotherapy/ (55195)
17 psychotherap$.mp. (204413)
18 Cognitive Behavioral Therap$.mp. (18892)
19 (cognitive adj2 therap$).mp. (45660)
20 behav$ therap$.mp. (52915)
21 Cognitive Behavior Therapy/ (21522)
22 14 or 15 or 16 or 17 or 18 or 19 or 20 or 21 (256324)
23 Mobile Applications/ (1390)
24 (mobile adj2 (app$ or therap$)).mp. (3475)
25 (mhealth or m-health).mp. (1468)
26 wearable$.mp. (1759)
27 patient$ portal$.mp. (253)
28 text messag$.mp. (3219)
29 Text Messaging/ (1202)
30 (mobile or technology-assisted or computer-based or internet-based or information technology or web-based or technology-mediated or technology-enabled).mp. (59805)
31 virtual realit$.mp. (11545)
32 Virtual Reality/ (9420)
33 Telemedicine/ (6113)
34 (teletherap$ or telepsychiatr$ or telemed$ or telehealth or tele-therap$ or tele-psychiatr$ or tele-med$ or tele-health).mp. (8847)
35 telemental$.mp. (390)
36 teletherap$.mp. (147)
37 video game$.mp. (7240)
38 Computer Games/ (8191)
39 digital tool$.mp. (632)
40 23 or 24 or 25 or 26 or 27 or 28 or 29 or 30 or 31 or 32 or 33 or 34 or 35 or 36 or 37 or 38 or 39 (89818)
41 (dropout$ or drop out$).mp. (16245)
42 adheren$.mp. (34703)
43 ((user$ or patient$ or caregiver$ or care giver$) adj2 (engag$ or experience$)).mp. (27582)
44 uptak$.mp. (17693)
45 accept$.mp. (156735)

46 usability.mp. (6319)
47 41 or 42 or 43 or 44 or 45 or 46 (249045)
48 7 or 13 or 22 (691161)
49 40 and 47 and 48 (2158)

****

CINAHL

#	Query	Results
S1	(MH "Mental Disorders")	62,628
S2	(MH "Mental Health")	45,158
S3	MH mental health services	35,524
S4	(mental* ill* or mental* disorder* or mental* health*)	222,086
S5	(psychiatric N3 (client* or patient* or inpatient* or service* or treat* or program*))	30,127
S6	S1 OR S2 OR S3 OR S4 OR S5	235,927
S7	(MH "Suicidal Ideation") OR (MH "Suicide")	26,654
S8	suicide	38,919
S9	(MH "Hallucinations")	3,320
S10	Hallucinat*	5,523
S11	(MH "Crisis Intervention")	4,333
S12	crisis interven*	5,020
S13	(MH "Psychotherapy")	22,880
S14	psychotherap*	43,126
S15	Cognitive Behavioral Therap* OR cognitive N2 therap* OR behave* therap*	26,132
S16	(MH "Cognitive Therapy")	19,977
S17	S7 OR S8 OR S9 OR S10 OR S11 OR S12 OR S13 OR S14 OR S15 OR S16	114,101
S18	(MH "Mobile Applications")	9,605
S19	(mobile N2 (app* or therap*))	12,471
S20	(mhealth or m-health)	2,265
S21	wearable*	5,041
S22	patient* portal* OR text messag* OR ((mobile or technology-assisted or computer-based or internet-based or information technology or web-based or technology-mediated or technology-enabled))	87,030
S23	(MH "Patient Portals")	165
S24	(MH "Text Messaging")	3,467
S25	(MH "Virtual Reality")	5,941
S26	virtual realit*	8,734
S27	(MH "Telemedicine")	13,948
S28	(teletherap* or telepsychiatr* or telemed* or telehealth or tele-therap* or tele-psychiatr* or tele-med* or tele-health) OR telemental* OR teletherap*	29,059
S29	(MH "Video Games")	5,053
S30	video game* AND digital tool*	18
S31	S18 OR S19 OR S20 OR S21 OR S22 OR S23 OR S24 OR S25 OR S26 OR S27 OR S28 OR S29 OR S30	126,355
S32	dropout* or drop out*	10,024
S33	adheren*	68,687
S34	((user* or patient* or caregiver* or care giver*) N2 (engag* or experience*)).	64,560
S35	uptak*	46,341

S36	accept$	8,773
S37	usability	7,008
S38	S32 OR S33 OR S34 OR S35 OR S36 OR S37	199,091
S39	S6 OR S17	322,982
S40	S31 AND S38 AND S39	1,007

Web of Science

#	Query	Results
1	(((((TS=(mental disorders)) OR TS=(mental health)) OR TS=(mentally ill persons)) OR TS=(mental health services)) OR TS=((mental$ ill$ or mental$ disorder$ or mental$ health$))) OR TS=((psychiatric adj3 (client$ or patient$ or inpatient$ or service$ or treat$ or program$)))	405,192
2	((((TS=(Suicidal Ideation)) OR TS=(Suicide)) OR TS=(suicid$)) OR TS=(Hallucinations)) OR TS=(Hallucinat$)	120,391
3	(((TS=(Crisis Intervention)) OR TS=(crisis interven$)) OR TS=(Psychotherapy)) OR TS=(psychotherap$)	92,788
4	(((TS=(Cognitive Behavioral Therap$)) OR TS=(Cognitive Behavioral Therapy)) OR TS=(cognitive NEAR/2 therap$)) OR TS=(behave$ therap$)	58,177
5	(((#1) OR #2) OR #3) OR #4	616,863
6	((((((((((((((((TS=(Mobile Application)) OR TS=((mobile adj2 (app$ or therap$)))) OR TS=((mhealth or m-health))) OR TS=(wearable$)) OR TS=(Patient Portals)) OR TS=(patient$ portal$)) OR TS=(text messag$)) OR TS=(Text Messaging)) OR TS=((mobile or technology-assisted or computer-based or internet-based or information technology or web-based or technology-mediated or technology-enabled))) OR TS=(Virtual Reality)) OR TS=(virtual realit$)) OR TS=(telemedicine)) OR TS=((teletherap$ or telepsychiatr$ or telemed$ or telehealth or tele-therap$ or tele-psychiatr$ or tele-med$ or tele-health))) OR TS=(telemental$)) OR TS=(teletherap$)) OR TS=(Video Games)) OR TS=(video game$)) OR TS=(digital tool$)	1,236,625
7	(((((TS=((dropout$ or drop out$))) OR TS=(adheren$)) OR TS=(((user$ or patient$ or caregiver$ or care giver$) adj2 (engag$ or experience$)))) OR TS=(uptak$)) OR TS=(accept$)) OR TS=(usability)	1,010,632
8	((#5) AND #6) AND #7	1,527
